# Tradeoffs and constraints on the evolution of tailocins

**DOI:** 10.1016/j.tim.2024.04.001

**Published:** 2024-06-18

**Authors:** Talia Backman, Hernán A. Burbano, Talia L. Karasov

**Affiliations:** 1 School of Biological Sciences, University of Utah 257S 1400E, Salt Lake City, UT 84112, USA; 2 Centre for Life’s Origins and Evolution, Department of Genetics, Evolution, and Environment, University College London, London, UK

## Abstract

Phage tail-like bacteriocins (tailocins) are protein complexes produced by bacteria with the potential to kill their neighbors. Widespread throughout Gram-negative bacteria, tailocins exhibit extreme specificity in their targets, largely killing closely related strains. Despite their presence in diverse bacteria, the impact of these competitive weapons on the surrounding microbiota is largely unknown. Recent studies revealed the rapid evolution and genetic diversity of tailocins in microbial communities and suggest that there are constraints on the evolution of specificity and resistance. Given the precision of their targeted killing and the ease of engineering new specificities, understanding the evolution and ecological impact of tailocins may enable the design of promising candidates for novel targeted antibiotics.

## Tailocins and microbe–microbe competition

A bacterium must compete with its microbial neighbors to survive. Because of this strong selection, bacteria have evolved hundreds of diverse tactics for regulating their competitors [1], from sequestering resources away from neighbors [2] to producing toxins that will directly kill neighbors [3]. Among the most exquisitely tailored of these mechanisms is the tailocin, which is capable of targeting subsets of closely related strains while leaving other close relatives unharmed. Potent antibiotics, tailocins are distributed broadly across Gram-negative bacteria and are found in several Gram-positive bacteria [4–6]. With their specific targeting capacity and evolvability, tailocins have the capacity to act as significant architects of microbiome composition and show promise in the engineering of new antibiotics. They are also common, found in the microbiomes of both host-associated [7] and non-host-associated [8] environments. Their frequency and precision beg the question: in natural microbial communities, do tailocins play a significant role in structuring the surrounding microbiota?

In this review, we outline and discuss what is known about the role of tailocins in microbial ecology. We first provide an overview of tailocin mechanisms of specificity and killing (reviewed thoroughly in [9–12]) and then summarize the state of knowledge on their ecology and evolution in bacterial populations. We point out that our current understanding of tailocin dynamics is limited primarily to *in vitro* experimentation. While comparative genomics has revealed a vast genetic diversity and rapid turnover at tailocin loci, the competitive interactions that drive this evolution remain unexamined. Whether tailocins are important weapons that shape microbial community structure or just tinkerers on the margins remains an open question.

## The basics of tailocin biology

**Figure 1.**
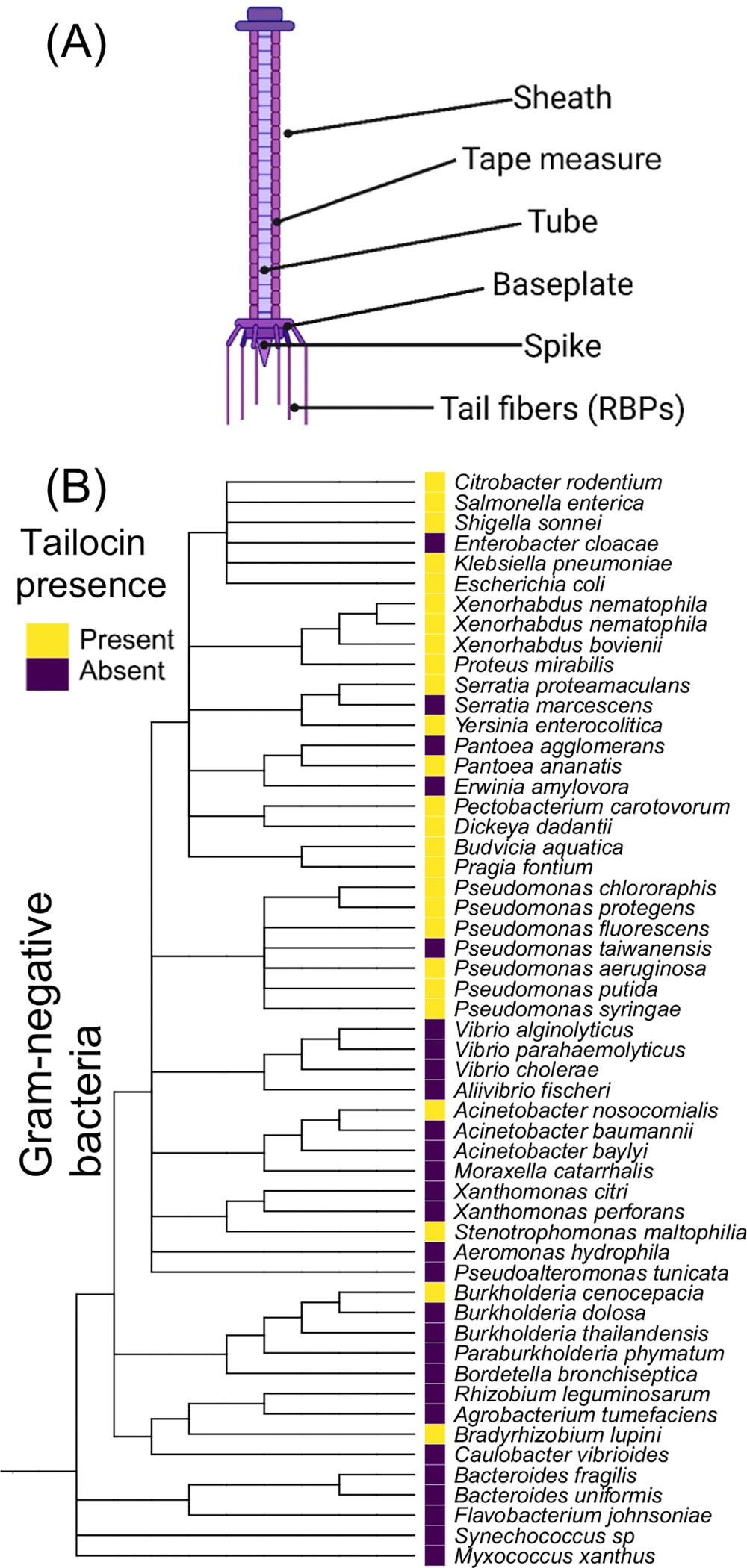
Structure of R-type tailocins and their phylogenetic distribution in Gram-negative bacteria. (A) Morphology of an R-type tailocin. (B) Phylogeny of representatives of Gram-negative bacteria assembled with the National Center for Biotechnology Information (NCBI) common tree tool [74]. The presence (yellow) and absence (purple) of tailocins were ascertained based on literature reports. Abbreviation: RBPs, receptor-binding proteins.

**Figure 2.**
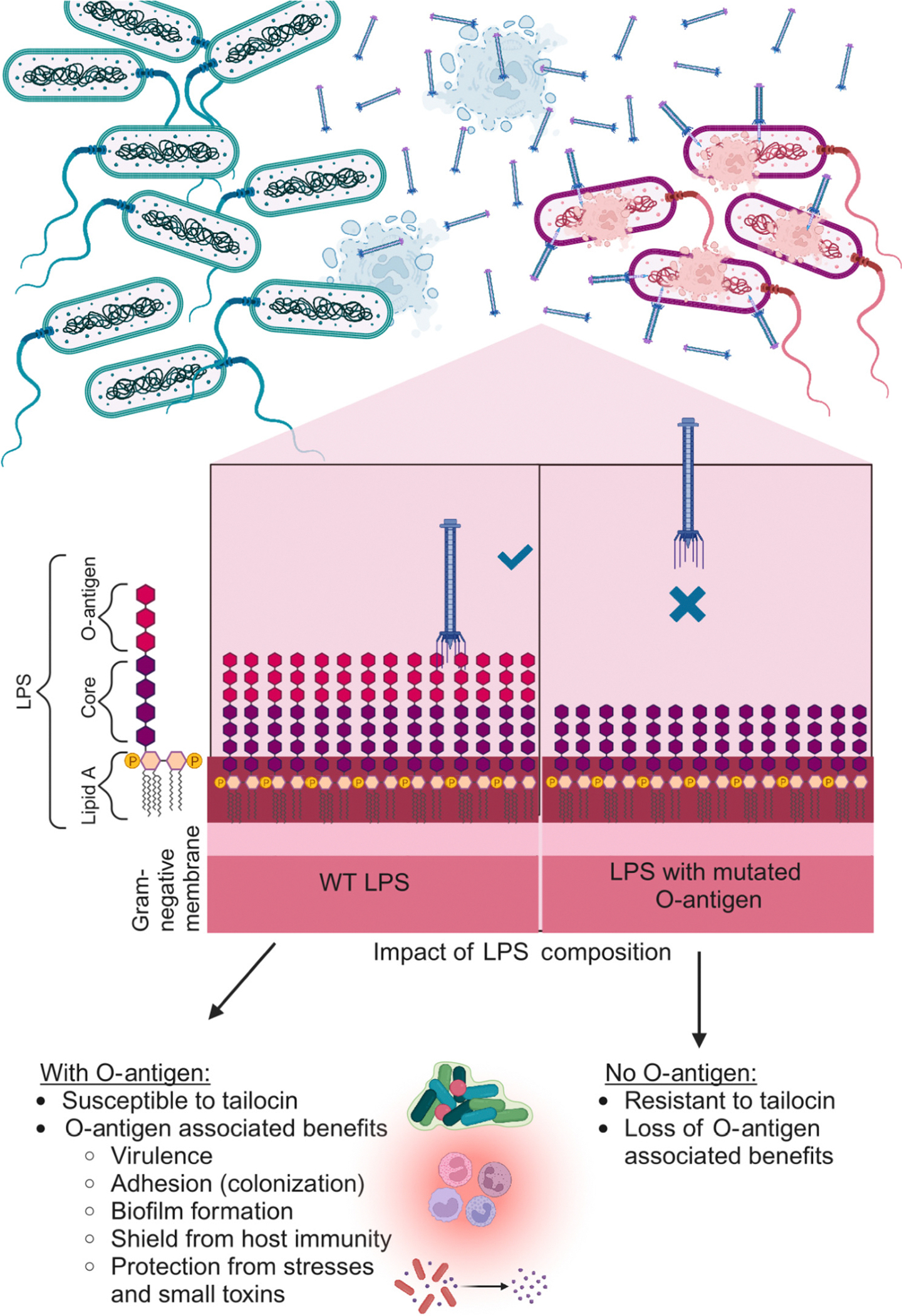
Tailocin killing specificity follows a lock-and-key model, and evolving tailocin resistance comes with tradeoffs. Schematic illustration of tailocin producers (blue) killing sensitive cells (pink). The mechanism for killing specificity is a lock-and-key model where tail fibers in the tailocin must be able to bind to polysaccharides in the target lipopolysaccharide (LPS). The check mark indicates successful tail fiber binding, whereas the cross indicates tail fibers that are unable to bind. Changes to the LPS structure confer resistance to tailocins. However, LPS is involved in diverse physiological processes and modifications to the LPS may negatively influence these other processes such as adhesion to surfaces, biofilm formation, and avoidance of the host immune system. Abbreviation: WT, wild type. The figure was created with BioRender.com.

Tailocins are protein complexes produced by bacteria that can target and kill neighboring bacteria. Tailocins share a common ancestor with phages [9], but have repurposed the phage machinery for host-cell targeting for their own fitness advantage (Figure 1A). Importantly, the current understanding is that a wild-type bacterium is immune to its own tailocin (but see the rare exception in [13]) and because of this self-immunity can release tailocins into the surroundings, suppress non-self, while leaving its own genetic lineage unaffected (Figure 2) [14,15]. The targeting of the tailocin is tunable, with single gene additions or gene swaps shifting killing specificity [16,17].

There are two major classes of tailocins – the F and R types. R-type tailocins consist of a rigid contractile tail-like structure, while the non-contractile F-type tailocins exhibit greater flexibility. When an R-type tailocin binds to a target cell, its sheath and tube contract, causing the inner tube to insert into the membrane forming a channel, leading to depolarization of the membrane and death of the target cell [11,18–21]. Work in the 1960s on tailocins used different ratios of tailocins and a target *Pseudomonas aeruginosa* strain to estimate the killing capacity of a tailocin complex [22–24]. This work showed that a single producing cell could release hundreds of tailocin molecules. These numbers likely differ between tailocin systems, though the potency of tailocin killing remains uncharacterized for most systems.

The production of tailocins (and also prophages) is triggered by diverse stresses, including induction of the SOS response [25,26]. Exogenous triggers of the SOS response include UV light, drugs, oxidants, and chemical mutagens, while endogenous triggers include metabolic intermediates, stalled replication forks, and defects following recombination and chromosome segregation [27]. Both the endogenous and exogenous triggers result in the presence of single-stranded DNA, which initiates the SOS response [28]. While tailocin producers induce tailocins in the presence of competitor bacteria [29], the specific trigger of the SOS response under these conditions remains unclear. Single producer cells produce tailocins at the center of the cell, transport the tailocins to the cell poles, and eject the tailocins over several tens of micrometers into the environment through cell lysis, where they will possibly come into contact with sensitive competitor cells [29]. Killing by tailocins happens quickly, with killing of non-self neighbors occurring within 3 h of exposure [29]. In the laboratory, studies that tested purified tailocin on panels of target strains have repeatedly shown that the most common susceptible targets of a tailocin are closely related bacteria, oftentimes bacteria of the same species or genus [7,14,15,29]. Tailocins can target more distantly related species [30,31], but this divergent targeting thus far seems to be more the exception than the rule. Most studies are limited in testing to close relatives, however, so this same pattern could also be the result of a sampling bias.

## Tailocins and bacteriophages share a common evolutionary origin

Tailocins represent only one of the numerous categories of contractile injection systems [12] sharing homology with phage components. Bacteria employ these complexes to carry out vital biological functions that bolster their adaptability and competitive advantage over other bacteria [1,32]. The extensively researched type 6 secretion system (T6SS) operates as a needle-like injection apparatus, capable of breaching the cell envelopes of both prokaryotic and eukaryotic cells, thereby facilitating the delivery of effector proteins [33], whereas phage tail-like particles are capable of secreting proteins and thus have been dubbed ‘toxin-delivering tailocins’ [34]. Tailocins, the T6SS, and phage tail-like particles are evolutionarily linked to phages, sharing a common set of core proteins [10]. However, in contrast to phages, they all are ‘headless’, denoting the absence of the DNA-containing capsid as well as genes for head formation, nucleic acid replication, and packaging. Except for the F-type tailocins, which bear a resemblance to the tails of siphophages, R-type tailocins, T6SS, and phage tail-like particles share homology with phages of the family *Myoviridae* [35–37].

Tailocins have likely evolved independently many times. The phylogenetic grouping of tailocins alongside fully functional phages can serve as an indicator to determine whether tailocins have originated from single or multiple independent events. For instance, R-type and F-type tailocins of *Pseudomonas* share an ancestral origin with P2 and λ phages, respectively [37]. Remarkably, not all R-type tailocins stem from a singular evolutionary occurrence; instead, they represent independent co-options by phages. This is exemplified by the molecular and genomic convergence of independently acquired R-type tailocins within the genomes of *Pseudomonas syringae* (derived from phage Mu) and *P. aeruginosa* (derived from phage P2) [38]. Furthermore, a phylogenetic examination of sheath proteins, jointly present in both phages and R-type tailocins, revealed a clustering of R-type tailocins with different phage species. This discovery reinforces the concept of multiple origins for tailocins, or in simpler terms, the existence of diverse routes by which host bacteria ‘domesticate’ these phage-derived tails [9].

## Tailocins are widespread across Gram-negative bacteria

Like tailocins, the T6SS also shares a common ancestor with phages and functions in inter-strain competition. A previous computational survey found T6SS in more than 25% of Gram-negative bacterial genomes [39]. No such comprehensive analysis has been conducted to date for tailocins, but it is clear that tailocin genomic clusters are broadly distributed in Gram-negative bacteria (Figure 1B [1,37,40]). Both R-type [4] and F-type [4] tailocins have been identified in Gram-positive bacteria.

## Tailocin specificity is conferred by the tail fiber, and resistance to tailocins by the target lipopolysaccharide

Tailocins are strain-specific killers. What makes a bacterium resistant or susceptible to a given tailocin? In Gram-negative bacterial interactions, the tail fibers of tailocins (also known as a class of receptor-binding proteins – RBPs) bind to the outer membrane of the target cell, specifically to components of the lipopolysaccharide (LPS) outer membrane [14,30,41–43]. Tail fiber binding to LPS is a ‘lock-and-key’ model by which a tailocin must have a tail fiber that is able to bind, and the target cell must have an accessible outer membrane to which the receptor binds. Strains are known to be resistant to self-intoxication from their own tailocin due to encoding an LPS and tailocin that do not bind one another [14]. Studies have found that thinning of the O-antigen density in LPS increases sensitivity to self-produced tailocins [14,44], suggesting that more dense LPS can shield important receptors from tailocins (Figure 2). Together, these findings suggest the LPS acts as either a receptor or a shield to tailocins. Other receptors of tail fibers for tailocins have not been identified to date, but there may be more to discover. A saturation mutagenesis screen of *de novo* resistance to tailocin in a *Pseudomonas* strain found genetic mutations outside of the LPS that confer resistance [14]. These loci have yet to be further studied.

The LPS of Gram-negative bacteria is docked in the outer membrane with three layers: lipid A, core oligosaccharide, and O-antigen [45–47]. For example, the LPS in *P. aeruginosa* PAO1 has three outer core membrane components: the A-band with a repeating fucose and mannose O-antigen chain, the B-band with a repeating D-rhamnose O-antigen chain, and an uncapped core. Three kinds of tailocins have binding sites on the glucose and rhamnose carbohydrate substrates of the uncapped core [44]. In *P. syringae*, it is also likely that tailocins bind to rhamnose [41,43] The results suggest the presence of several different receptors for tailocins though some of them are shared among species.

## Tailocin genomic loci evolve quickly and are important for bacterial fitness

The widespread distribution of tailocins throughout the bacterial phylogeny [10] supports their significance in enhancing bacterial fitness. Consistent with this finding, it has been shown that prophage genes that encode core phage-related functions are under strong purifying selection in the bacterial chromosome. This is presumably attributed to their adaptive importance within the bacterial host [48]. Nevertheless, the degree of conservation varies among genes within individual tailocin clusters. While the vast majority of genes that encode the tailocin structure and some assembly proteins are well conserved, genes encoding RBPs and their chaperones display high variability [49,50]. RBPs, like the tail fiber protein, directly interact with the targeted bacteria, thus playing a critical role in defining the killing spectrum of tailocins [32]. Typically, tailocin clusters are distinguished by the presence of a single tail fiber gene within each cluster, and it is generally expected to observe just one tailocin cluster in a given bacterial genome [11].

What underlies the prevalence of this genomic organization (limited to a single tailocin per genome and one tail fiber allele per tailocin) across different bacterial taxa? Considering the prevalence of prophages in bacterial genomes, and without accounting for any selective constraint, it is statistically reasonable to anticipate the presence of multiple tailocins in some bacterial genomes. In theory, this could broaden the spectrum of bacterial strains targeted by a specific bacterium. Nevertheless, if the expression of tailocin clusters is not carefully regulated, having multiple tailocins in a genome might lead to interference between tailocin clusters during their assembly, in the same way as phage infections in a given bacterium may be convoluted by the expression of its resident prophages [48]. Rhizosphere-inhabiting pseudomonads might have already overcome this particular challenge, since the characterization of different strains of rhizospheric *Pseudomonas chlororaphis* revealed a high prevalence of a tailocin cluster that encodes two different tailocins [51]. Although these two tailocins are highly divergent from each other, and have distinct evolutionary origins, their secretion is facilitated by a single shared lysis cassette. The evolutionary divergence of the *P. chlororaphis* tailocins is also reflected in their different killing spectra, showing how the co-option of this ‘hybrid’ tailocin cluster broadens the killing spectrum of *P. chlororaphis* [51]. The substantial divergence between the two *P. chlororaphis*-encoded tailocins is likely responsible for preventing interference during their assembly, which has resulted in the maintenance of a single lysis cassette. Moreover, the detailed analysis of one of the *P. chlororaphis* tailocins revealed the presence of three different tail fiber genes, which are assembled in three distinct tailocin complexes and further expand the range of organisms targeted by *P. chlororaphis* [7].

The presence of tailocin clusters with more than one tailocin and of tailocins with more than one tail fiber protein appears to be a distinguishing trait among Gram-negative rhizospheric bacteria, including strains of *P. chlororaphis* and *Kosakonia radicincitans* [7,11]. Multiple strain specificities can be produced through reversible genomic reorganization within a strain. A tailocin in strains of *Erwinia carotovora* encodes an invertible tail fiber region alongside an invertase. Inversion of the locus happens frequently in the cell population, and the inverted tail fiber haplotypes confer different killing spectra [52,53]. It stands to reason that bacteria hosting tailocins with a more diverse genomic organization and, consequently, a broader killing spectrum, will flourish within the highly diverse and likely very competitive microbial community found in the rhizosphere.

## Coevolution of tailocins and their targets maintains genetic diversity

Thus far we have discussed the mechanisms of tailocin killing and the molecular evolution of the tailocin and its targets. What evolutionary forces promote the diversification and maintenance of tailocins?

Tailocin diversity is evident not only through differences in the genomic frequency and organization of tailocin clusters but also at the intraspecific level. In both *P. syringae* and wild *Pseudomonas viridiflava* isolates, genes responsible for crucial aspects of bacterial targeting, such as RBPs, including the tail fiber protein and its chaperone, show higher variability compared with other components within the tailocin gene cluster [49,50]. In both species, phylogenetic trees built from RBPs are discordant from those built from either other tailocin cluster genes or the bacterial core phylogeny. This phylogenetic discordance suggests that RBPs might have been acquired by localized recombination [49,50]. Although the mechanisms underlying this remarkably precise recombination process remain unidentified at present, it is evident that recombination greatly contributes to the high level of intraspecific genetic diversity of RBPs.

The high intraspecific genetic diversity of RBPs is also apparent in the site frequency spectrum, a summary of the distribution of allele frequencies in a given set of loci across a population. A preliminary analysis of the site frequency spectrum of RBPs in wild populations of *P. viridiflava* using the summary statistic Tajima’sD [54] revealed an excess of intermediate frequency polymorphisms [50]. These polymorphisms are likely the outcome of mutations accumulating over extended timescales on different RBP variants, indicated by the positive Tajima’s D values. Balancing selection might be the evolutionary force that maintains diverse tailocin variants over time (but see the next section). Furthermore, the maintenance of genetic diversity in RBPs is supported by a preliminary analysis of historical *P. viridiflava* genomes derived from century-old herbarium specimens, which revealed that a defined set of tailocin variants have circulated in the same bacterial metapopulations for almost 200 years [50]. This observation might imply a restricted range of mechanisms that govern tailocin sensitivity and specificity.

## Whether a tailocin is adaptive depends on the microbial ecology

Beyond the capacity to kill specific bacteria, what is the influence of tailocins on the larger bacterial community? Tailocins are distinguished by a unique set of molecular characteristics that potentially impact their role in microbial ecology. Tailocins are mechanical weapons that are released by cell lysis, enabling them to exert their lethal effects in a contact-independent manner [1]. This is in contrast to T6SS, which requires direct contact between neighboring bacterial cells [33]. Therefore, in principle, tailocins can exert a long-range influence on the configuration of microbial communities. Whether deploying a long-range weapon or short-range weapon is better may depend on the ecology of the producing bacterium. Booth *et al.* [55] modeled how the abundance of the tailocin-producing strain in a microbial community affects the optimal strategy for killing. Both their theoretical models and experimental testing in *P. aeruginosa* predicted that long-range weapons such as tailocins require high frequencies and high cell densities to be effective. By contrast, contact-dependent mechanisms such as the T6SS substantially improve the fitness of the producing strain even when at low frequencies. In other words, these results predict that tailocins are ecologically useful for those bacterial strains that are abundant.

Whereas bacteria exhibit resistance when encountering their self-produced tailocins in the environment, it is worth noting that tailocins are released as a result of cell lysis, leading to the death of the producing bacterial cell. This implies a division of labor among bacteria, with clonemates, rather than individual cells, serving as units of selection, as only a certain portion of bacterial cells sacrifice themselves to protect their fellow clonemates [1]. This sharply contrasts with the conventional bacterial toxin–antitoxin system, in which toxin secretion does not result in the death of the producing cell. Instead, the cell is shielded by an antitoxin that either inhibits toxin expression or sequesters it in a complex [56]. What ecological factors trigger the production of tailocins and determine the fraction of self-sacrificing clonemates?

The expression by a bacterium of its antibacterial weaponry is thought to occur in response to bacterial perception of ecological competition rather than in the presence of abiotic stress [57]. Bacterial density can serve as an indicator of a competitive microbial community. For instance, the production of tailocins by a strain of *P. chlororaphis* confers an important competitive strategy for bacterial persistence in the densely populated microbial communities of the rhizosphere [7]. Tailocins also play a pivotal role in determining the ‘winning’ strain in biofilms formed by a *P. aeruginosa* strain isolated from the lungs of patients with cystic fibrosis [58]. Biofilms are densely populated communities of bacteria that adhere to each other and are typically associated with a surface. Traditionally, they have been regarded as cooperative endeavors, but it has been postulated that they evolve in response to ecological competition driven by bacterial weaponry [59]. The regulation of tailocin production extends beyond competitive conditions; however, –tailocin-encoding genes are upregulated in plant infections with *P. syringae* even in the absence of competing strains [60].

The typically limited killing spectrum of tailocins, where they target bacteria closely related phylogenetically to the producing strain [14], might already suggest that tailocins are deployed against bacteria occupying highly similar ecological niches. Conversely, the range of tailocin haplotypes within a specific bacterial metapopulation, determined by the diversity of RBPs, could potentially result from a higher frequency of horizontal gene transfer (HGT) among closely related bacteria. Notably, investigations into the intraspecific diversity of RBPs in *P. syringae* and *P. viridiflava* populations show discordant phylogenies that are likely the result of HGT [49,50]. There are few characterized examples of tailocins that can eliminate distantly related strains [31,50], highlighting that the modularity of tailocin components allows for the incorporation of a broad range of RBPs. This suggests that the evolution of the tailocin killing spectrum is not structurally constrained but rather restricted by the diversity of coexisting bacteria, which are the source of RBPs.

## Evolving resistance to a tailocin comes with tradeoffs

Resistance to a given tailocin can evolve quickly simply through the modification of single components of LPS. One consequence of this rapid evolution is that a tailocin variant that was potent in one generation could quickly lose killing activity a few generations later. This scenario would result in rapid turnover of the tailocin variants that are the best killers, and thereby selected for in the population. Put more simply, because resistance can evolve quickly, the adaptive ‘half-life’ of a tailocin variant should be short. This straightforward expectation was not validated by recent surveys of tailocin evolution. Instead, in the *Pseudomonas* populations colonizing European plants, the same tailocin variants persisted for nearly 200 years and are still found globally [50]. New, simple resistances, can arise in the course of a few days but these new variants do not show evidence of persisting.

Why is turnover of tailocin variants seemingly so limited (at least in this system)? One plausible answer is that evolving resistance to a tailocin variant comes with pleiotropic costs to the bacterium. In other words a tradeoff. Indeed, LPS resistance mutations are likely to affect other bacterial functions negatively. For example, mutations of LPS that confer resistance to phages frequently render the evolved strain more sensitive to antibiotics (see [61] for review). Within a host, O-antigens that make a bacterium sensitive to a tailocin may alternately be important in shielding the bacterium from a host immune system. In an insect colonization model, Heiman *et al*. [42] found the tailocin-resistance mutations in a *Pseudomonas* strain had a negative fitness effect upon colonization – the insect immune system recognized the tailocin-resistant strain (with a mutated LPS) better than it did the susceptible strain. The consequence is a tradeoff between evolving complete resistance to tailocins and colonizing the insect host [42] (Figure 2). Hockett *et al*. [62] demonstrated tradeoffs in resistances between a tailocin and another toxin – they found that loss of O-antigen in a *Pseudomonas* strain conferred resistance to a common R-type tailocin, but *de novo* sensitivity to a co-occurring S-type bacteriocin. Returning to the question of the limited turnover of tailocin variants over time, one possibility is that most tailocin variants are accompanied by tradeoffs that outweigh their fitness benefit in competitive environments. Only a subset of the variants, perhaps only a few, are sufficiently adaptive to be maintained in a given population.

The maintenance of diverse tailocin variants within wild bacterial populations suggests that the balance between the aforementioned tradeoffs varies across time and/or space – at some points in time, it is more important to defend against tailocins, and at other points in time, it is more important to have a thick LPS to shield against host toxins, for example [42]. Gómez *et al.* [63,64] demonstrated in a phage selection system that selection for resistance both fluctuated over time and was influenced by the nutrient availability of the system. Given the similarities in LPS-mediated resistance mechanisms to phages and tailocins, it is easy to speculate that selection for tailocin resistance can similarly fluctuate.

**Figure 3.**
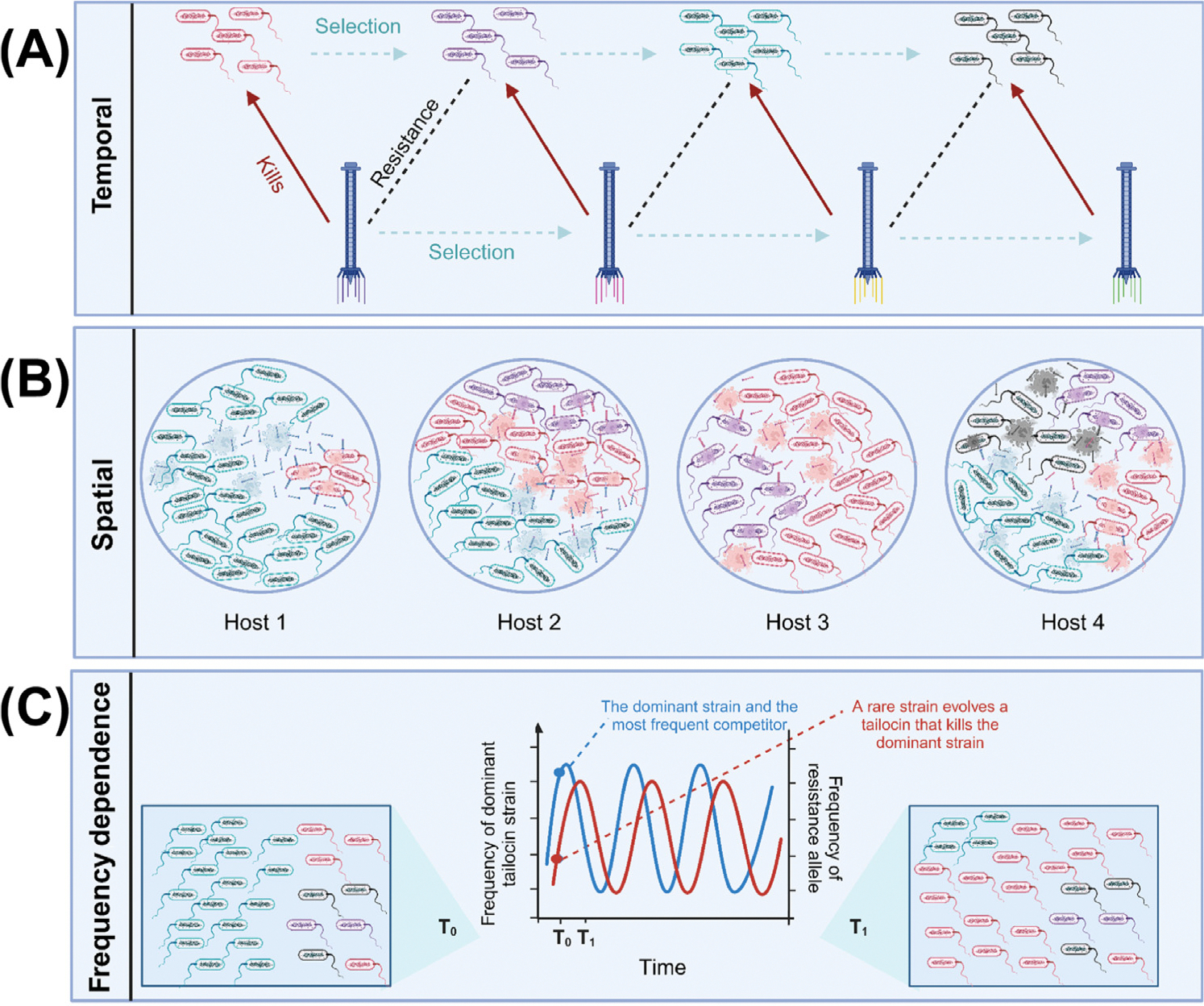
The evolutionary and ecological forces that maintain tailocin diversity in host populations. Several selective forces can maintain tailocin diversity within populations. With temporal variation (A) in selection, the tailocin variant that is advantageous will change throughout time. Spatial differences between hosts and environments (B) will lead to differences in neighboring competitors across space. Negative frequency-dependent selection (C) is another scenario that can maintain diversity in populations over time. With this selection, the adaptive advantage of an allele decreases with the frequency of the allele. Frequency dependence graph adapted from concepts in Stahl *et al*. [65]. This has not yet been observed in tailocin populations to date but has been observed in other host–pathogen systems [75]. The figure was created with BioRender.com.

Similar to other forms of antagonistic biotic interactions [65], it is plausible that balancing selection preserves genetic diversity critical for bacteria–bacteria competition. In general, balancing selection maintains beneficial genetic and phenotypic variation within populations. However the relative fitness of alleles under balancing selection changes with time, space, or population frequency (Figure 3). Thus, the preservation of genetic diversity by balancing selection can result from several distinct adaptive mechanisms [66], including selection that varies across time and space in a panmictic population [67] and negative frequency-dependent selection.

When selection varies temporally (Figure 3A) or spatially (Figure 3B) within a panmictic population, the diversity of selection coefficients across time or different regions of a species or population range contributes to the persistence of alleles that confer benefits only at particular times or in specific locations [67,68]. At each of these times or specific locations, the potential for positive frequency-dependent selection exists. This type of selection will act to reduce diversity temporally or locally, but temporal and spatial separations between populations can lead to a patchwork pattern where certain genotypes dominate temporarily or only in their immediate vicinity and not across broader regions [1]. The relevant scale at which tailocins exert their killing activity, along with the spatial distribution and density of distinct tailocin variants, is yet to be explored. Characterization of the spatial distribution of tailocin variants has the potential to unveil the spatial ecology and the factors contributing to the preservation of a diverse array of tailocin variants within microbial communities.

Finally, in negative frequency-dependent selection, the fitness of an allele is inversely correlated with its abundance in the population [67,69] (Figure 3C). A rare tailocin can enhance the success of its producer since, in the absence of significant selective pressure, it is improbable that the surrounding microbial community harbors an LPS conferring resistance to an uncommon tailocin variant. If tailocin producers and their bacterial targets are engaged in mutual evolution, negative frequency-dependent selection can drive the dynamics akin to the Red Queen hypothesis, in which species have to constantly evolve to survive [69].

## Can tailocins be harnessed to develop strain-specific antibiotics?

Tailocins have clear potential for the development of novel antibiotics. Synthetic antibiotics largely target broad classes of bacteria resulting in unintended suppression of the whole microbiome [70]. By contrast, tailocin killing can be specific and targeted. The lock-and-key logic of the tail fiber–LPS interaction has lent itself to the engineering of new targets simply by swapping tail fibers between tailocins or adding phage targeting proteins to tailocins [71]. Proof-of-concept studies demonstrated that tailocins can be used in animal and plant models to manipulate the resident microbiota. In tomato, Príncipe *et al.* [72] tested whether pretreatment with a tailocin from *Pseudomonas fluorescens* could suppress subsequent infection with the plant pathogen *Xanthomonas vesicatoria*. Tailocin pretreatment led to a dramatic 39% reduction in disease incidence. Similar suppression was observed for *P. syringae* pathogens [73]. Preliminary results indicate that tailocins can also be used to treat infections in animals. Gebhart *et al.* [17] modified a *Clostridium difficile* tailocin with the tail fiber of a *C. difficile* bacteriophage. Oral administration of the tailocin in mice suppressed *C. difficile* infection in the mouse gut. The logistics of the development of tailocin antibiotics remain largely unexplored.

## Concluding remarks

Host health is frequently associated with a microbial community that is diverse and stable. Tailocins, a broadly distributed and targeted mechanism of bacterial competition, may provide a mechanism of resistance to pathogenic clonal spread [50] (see Outstanding questions). These features make tailocins good candidates for architects of microbiome composition and health. While several studies have demonstrated the potential for tailocins to shape the structure of surrounding microbial communities, their ecological role in the natural environment remains largely uncharacterized. To understand the importance of tailocins, and their potential in bioengineering, more research is needed on their activity and evolution in natural settings.

## Highlights

Tailocins, phage tail-like bacteriocins, are bacterial protein complexes that kill neighboring bacteria, thereby suppressing competitors.

The specificity of tailocins depends on their tail fibers, which bind to the target cell’s outer membrane, especially binding to specific lipopolysaccharide (LPS) components in a lock-and-key manner. This LPS binding defines the range of bacteria that tailocins can kill.

Tailocin genomic loci exhibit differential rates of evolution, with conserved genes involved in structural components and variable genes, such as the tail fibers, determining specificity.

The maintenance of diverse tailocin variants over time is likely driven by balancing selection, contributing to long-term persistence within bacterial populations.

A tradeoff exists between evolving resistance to tailocins and colonizing the host.

Tailocin specificity can be easily modified by swapping tail fibers.

## Outstanding questions

Why do tailocins primarily target closely related bacteria?

Why do bacteria typically encode only one (or two) tailocins? Why not target more bacteria?

What are the major tradeoffs to evolving broadly resistant LPS?

What determines the proportion of bacteria that self-sacrifice and produce tailocins? (How do bacteria regulate tailocin-mediated self-sacrifice?)

Which ecological factors within microbial communities promote the evolution of tailocins?

What are the timescales of tailocin maintenance in natural populations?

For competition within a microbial population, how long is a particular tailocin useful before the competitors evolve resistance?
